# Reply to: Ultrasonographic assessment of the muscle mass of the rectus femoris in mechanically ventilated patients at intensive care unit discharge is associated with deterioration of functional status at hospital discharge: a prospective cohort study

**DOI:** 10.62675/2965-2774.20260387

**Published:** 2026-03-03

**Authors:** Thiele Cabral Coelho Quadros, Thaline Lima Horn, Marina Santos de Moraes, Luisa da Cunha Selmo, Alexandre Ribas, Clarissa Blattner, Márcio Manozzo Boniatti

**Affiliations:** 1 Pontifícia Universidade Católica do Rio Grande do Sul Physiotherapy Department, Hospital São Lucas Porto Alegre RS Brazil Physiotherapy Department, Hospital São Lucas, Pontifícia Universidade Católica do Rio Grande do Sul - Porto Alegre (RS), Brazil.; 2 Universidade Federal do Rio Grande do Sul Hospital das Clínicas de Porto Alegre Porto Alegre RS Brazil Hospital das Clínicas de Porto Alegre, Universidade Federal do Rio Grande do Sul - Porto Alegre (RS), Brazil.

## To the Editor,

We read with interest the correspondence by Finsterer et al.^(1)^ regarding our paper on the relationship between the Barthel index (BI) and quadriceps muscle ultrasound measurements in patients who required prolonged mechanical ventilation.^(2)^ We are pleased to provide clarifications and additional context addressing the points raised.

We fully agree that multiple factors, including comorbidities, medications, nutritional status, pre-hospital physical condition, and duration of immobility influence muscle mass and functional recovery. In our study, we sought to account for clinically relevant variables that could plausibly affect functional status at hospital discharge. Accordingly, our multiple linear regression model included age, sex, Medical Research Council (MRC) score at awakening, Simplified Acute Physiology Score 3 (SAPS 3) score, intensive care unit (ICU) length of stay, BI before ICU admission, corticosteroid and neuromuscular blocker use, orthostatic capacity at ICU discharge, and both quadriceps thickness (TQ) and rectus femoris cross-sectional area (CSA) at ICU discharge. Among these, only the CSA of the rectus femoris remained independently associated with the Barthel Index at discharge. Therefore, although we recognize that other unmeasured variables may contribute to variations in muscle mass, our model incorporated several key factors likely to influence both ultrasound measurements and functional outcomes in this population.

Regarding the ultrasound methodology, TQ and the CSA of the rectus femoris were assessed using standardized techniques previously validated in critically ill patients. To measure TQ, the linear transducer was positioned transversely at two-thirds of the distance between the anterior superior iliac spine and the upper edge of the patella, and the perpendicular distance between the upper margin of the femur and the superficial fascia was measured, encompassing both the rectus femoris and vastus intermedius muscles. Three images were acquired, and their mean value was used for analysis. This approach is consistent with methodologies described by other authors,^(3-6)^ who demonstrated excellent interobserver reliability for quadriceps ultrasound measurements, even among assessors with varying levels of experience. For CSA measurement, the internal contour of the rectus femoris was delineated at the same anatomical level, excluding the fascia from the evaluation, in accordance with previously published protocols. This method allows simultaneous quantification of both muscle thickness and CSA within a reproducible and clinically meaningful plane. The term "highest point of the femur and superficial fascia" referred to the perpendicular distance between the femoral cortical surface—the point of most excellent acoustic reflection - and the superficial fascia of the quadriceps at the two-thirds thigh level, a landmark widely adopted in muscle ultrasound studies of critically ill patients.^(6)^

Concerning the number of measurements, the statement in our Methods section - "three images were obtained, and the results averaged for both measurements" - referred to the acquisition of three ultrasound images for each variable (TQ and CSA), followed by calculation of the mean of the three values. No measurement was excluded. All three images were included in the analysis to minimize random variation and improve reliability, as recommended in previous ultrasound studies in critically ill patients.^(4,7)^ Since no measurement was discarded, there was no separate analysis of variability or coefficient of variation among the three readings; instead, the mean value was used to represent each patient's measurement, following standard practice in similar research protocols.

Muscle strength was assessed using the MRC scale according to the standardized procedure proposed by De Jonghe et al.^(8)^ This protocol evaluates six bilateral muscle groups - shoulder abduction, elbow flexion, wrist extension, hip flexion, knee extension, and ankle dorsiflexion - each graded from zero to 5, yielding a total score ranging from zero to 60. A score below 48 was considered indicative of ICU-acquired weakness, as commonly adopted in studies of critically ill patients. We agree that muscle strength and muscle mass are related but distinct physiological constructs, and our study aimed to explore their complementary roles in predicting functional status at discharge.

Regarding temporal changes in TQ, we would like to clarify that there was no significant increase in quadriceps thickness between Day 1 and Day 5. The mean values (2.3 ± 0.6mm on Day 1 and 2.5 ± 0.8mm on Day 5) did not differ statistically, indicating only minor variation within the expected measurement variability. As discussed in the manuscript, previous studies have shown that the most pronounced reduction in quadriceps thickness occurs after the sixth day of ICU stay, when the catabolic phase resolves and protein degradation predominates. Our results are therefore consistent with the existing literature, which describes substantial muscle deterioration throughout the ICU course rather than in the first few days of admission.

We acknowledge the authors’ observation regarding the sample size. As stated in our manuscript, the target sample size was not achieved due to strict inclusion criteria and the challenges of serial ultrasound assessments in critically ill patients. We explicitly recognized this limitation in the Discussion section, noting that "the limited sample size hindered the evaluation of ultrasonographic muscle mass variables’ discriminatory power, preventing the establishment of a definitive cutoff point". Nevertheless, the statistical analyses performed met the assumptions required for linear regression, and the associations observed between rectus femoris CSA and functional status at discharge were consistent with previous reports in similar populations. While the results should be interpreted with caution, they remain informative and hypothesis-generating, reinforcing the need for further studies with larger cohorts.

We also appreciate the suggestion to compare ultrasound measurements with additional ICU outcomes and to report the reasons for ICU admission. However, the primary aim of our study was to explore the association between quadriceps ultrasound parameters and patients’ functional status at hospital discharge, rather than to evaluate broader ICU outcomes, such as mortality or mechanical ventilation duration. To minimize confounding, our multivariate model included several relevant variables reflecting illness severity and ICU course - such as SAPS 3 score, ICU length of stay, use of corticosteroids and neuromuscular blockers, and orthostatic capacity at ICU discharge. These variables indirectly capture much of the heterogeneity related to the cause and course of critical illness. Future studies with larger cohorts may further investigate how quadriceps ultrasound findings relate to other clinically relevant ICU outcomes across different diagnostic categories.

Finally, we thank the authors for their interest and constructive comments on our study. We agree that further research with larger samples and prospective designs is warranted to validate the use of rectus femoris CSA and quadriceps thickness as prognostic or outcome measures in critically ill patients. Our study was intended as an exploratory investigation to highlight the potential of bedside ultrasound for assessing functional status at discharge, and we believe it contributes to the growing body of evidence supporting the role of muscle ultrasound in critical care rehabilitation and prognosis.
